# Supplementary material for: Improving Sensitivity of the Digits-In-Noise Test Using Antiphasic Stimuli
Source: Ear Hear. 2020 Feb 24;41(2):442–50. doi: 10.1097/AUD.0000000000000775 (PMC7015780; doi:10.1097/AUD.0000000000000775)
Supplement: Supplementary file 1 [file aud-41-442-s001.pdf]

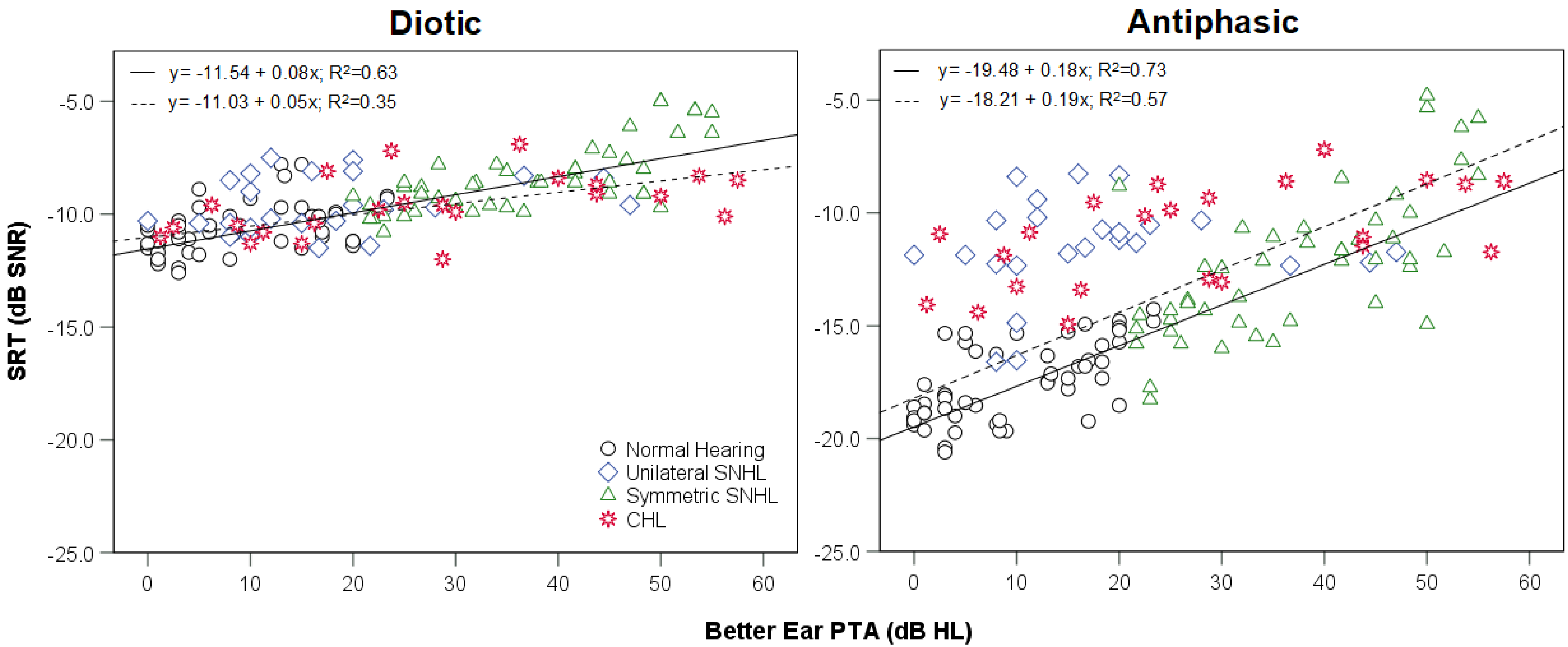

Figure. Correlations of the diotic and antiphasic DIN to better ear PTA. Solid lines are regression lines fitted to normal hearing and symmetric SNHL group data. Dashed lines are regression lines fitted to normal hearing and CHL group data. SRT; speech reception threshold, dB; decibel, SNR; signal to noise ratio, HL; hearing level.
